# Croupous and Catarrhal Pneumonia

**Published:** 1903-04-18

**Authors:** 


### CROUPOUS AND CATARRHAL PNEUMONIA.

IN a paper read before the St. George's Hospital Hunterian Society and published in the *Practitioner* for this month, Mr. S. Vere Pearson deals with the differential diagnosis between croupous and catarrhal pneumonia in infants. The question is an important one on account of the great frequency with which pneumonia occurs among infants, whether as a primary disease or as a complication occurring in the course of some other affection, and also because the prognosis will to a certain extent depend upon whether the pneumonia is of the croupous or catarrhal type. Croupous pneumonia is a single, well-recognised, acute specific disease, frequently occurring in infants, more frequently, Mr. Pearson thinks, than is generally recognised, and in which the prognosis is excellent. Broncho-pneumonia is not such a simple condition : it is divisible into several varieties, the most important grouping being into primary and secondary. As a general rule the two different kinds of pneumonia can be readily distinguished, though in some instances it may be impossible to differentiate between them. Croupous pnenmonia in the infant differs in few essentials from the same disease in an adult. It is characterised by its sudden onset, its continued high temperature and hurried respiration, its critical ending, and its rapid resolution. The consolidation of the lung is lobar, and is the result of fibrinous exudation, cells being relatively scarce in the exudate, and the walls of the bronchi being scarcely affected. The Fränckel-Weichselbaum diplococcus occurs in at least 90 per cent. of these cases. The physical signs develop rapidly and are well defined. Expectoration and rigors are not present in infants, but vomiting at the onset is common. Cough is seldom distressing, and laboured breathing, inspiratory dyspnœa, and cyanosis are absent, or, if present, are not prominent symptoms unless respiratory failure is commencing, when cyanosis may be marked. Prostration is great at an early stage of the illness, and as a rule steadily diminishes afterwards. Convalescence is rapid and the prognosis is good. In broncho-pneumonia the onset is more gradual ; the illness is often secondary to bronchitis, or one of the exanthems. The temperature, instead of remaining high as in croupous pneumonia, undergoes marked remissions ; the course is prolonged, resolution is slower, and the temperature falls by lysis. The signs of pneumonia are mingled with signs of bronchitis, and are slower to develop and often ill-defined. Cough is more constant and distressing than in croupous pneumonia. Cyanosis is common, and dyspnœa, often with some recession of the soft parts of the chest, is noticeable. Prostration is greater than in croupous pneumonia, and steadily increases. Convalescence is gradual, and the prognosis is bad. Relapses are not infrequent. The exudate is cellular rather than fibrinous, and the bacteria found are various ; the pneumococcus is the commonest, though not met with so constantly as it is in croupous pneumonia. The aspect and general condition of the patient are often the most important aids in the differential diagnosis, as they largely depend upon a difference in the character of the dyspnœa. In broncho-pneumonia there is inspiratory dyspnœa with laboured breathing and recession of the chest wall. The great rapidity of the respirations characteristic of croupous pneumonia may occur in broncho-pneumonia, but the dyspnœa possesses the quality just mentioned, which is seldom seen in lobar pneumonia.

